# Travel-Acquired Furuncular Cutaneous Myiasis Mimicking a Bacterial Abscess in a Pediatric Patient

**DOI:** 10.7759/cureus.103405

**Published:** 2026-02-11

**Authors:** Simran K Chandawarkar, Ibrahim Amjad

**Affiliations:** 1 College of Medicine, Northeast Ohio Medical University, Rootstown, USA; 2 Plastic Surgery, AmjadPlastics, Miami, USA

**Keywords:** bacterial abscess, botfly, dermatobia hominis, furuncular cutaneous myiasis, pediatric, travel-acquired infection

## Abstract

Furuncular cutaneous myiasis can closely mimic common bacterial abscesses, especially in regions where *Dermatobia hominis* is not endemic. Here, we present a case of a healthy six-year-old male who returned from a Caribbean cruise and developed four painful nodules on his scalp and arm that were initially presumed to be bacterial abscesses. Incision and drainage yielded little to no purulence. Operative exploration under general anesthesia revealed four intact *D. hominis* larvae, all of which were successfully extracted. This case underscores the importance of obtaining a travel history and considering myiasis when "abscesses" do not behave as expected, particularly when attempted drainage yields minimal material. Pediatric cases can present with multiple lesions, and definitive treatment may require operative removal in young patients who cannot tolerate awake extraction. Awareness of this entity is critical to avoid unnecessary antibiotics, repeated procedures, and delayed diagnosis in children presenting with furuncular lesions after travel to endemic areas.

## Introduction

Cutaneous myiasis is an infestation of human skin by fly larvae, most often presenting in a furuncular (boil-like) form. *Dermatobia hominis* (the human botfly) is endemic to Central and South America and is the leading cause of furuncular myiasis in travelers returning to the United States [1]. Transmission occurs via phoresy - adult botfly eggs are deposited on a vector insect (most commonly mosquitoes), which then delivers the larvae to human skin during a bite; the larvae penetrate the dermis, mature over several weeks, and eventually exit the host to pupate in soil [2]. The typical lesion is an erythematous nodule resembling a painful abscess, often distinguished by a central punctum with serosanguineous discharge and sometimes a visible larval breathing pore [1]. Patients may report a sensation of movement or intermittent pain within the lesion.

Myiasis is uncommon in the United States, and most cases are imported from tropical regions such as Central and South America. Furthermore, pediatric cases are very infrequently reported, especially in children without pre-existing wounds or travel to endemic areas [3]. Consequently, clinicians in non-endemic regions may not recognize furuncular myiasis, and lesions are often misdiagnosed as routine bacterial abscesses [4]. This can lead to inappropriate antibiotic use and repeated unsuccessful incision and drainage (I&D) attempts [4]. Here, we report a case of *D. hominis* myiasis in a child with multiple lesions initially mistaken for abscesses, highlighting the diagnostic challenge and importance of a travel history and awareness of this entity in pediatric patients.

## Case presentation

A previously healthy six-year-old male presented with one week of painful scalp and arm lesions. The patient noted three tender, erythematous nodules on the scalp and one on the right arm, all of which had enlarged over several days. He had no fever or systemic symptoms. Initial emergency department evaluation considered these lesions to be bacterial abscesses. Incision and drainage (I&D) was attempted on one lesion, but little to no pus was obtained. The child was started on empiric clindamycin therapy for a presumed staphylococcal infection and admitted for further management.

On hospital admission, multiple fluctuant "abscesses" were noted, but the lack of purulence raised concern for an atypical cause. Infectious disease was consulted, and upon detailed history, the family reported returning from a Caribbean cruise one week before lesion onset. Notably, the child had gone horseback riding on a farm in Belize, where *D. hominis* is endemic. No insect bites were reported, but the differential diagnosis broadened to include parasitic infections acquired abroad.

**Figure 1:**
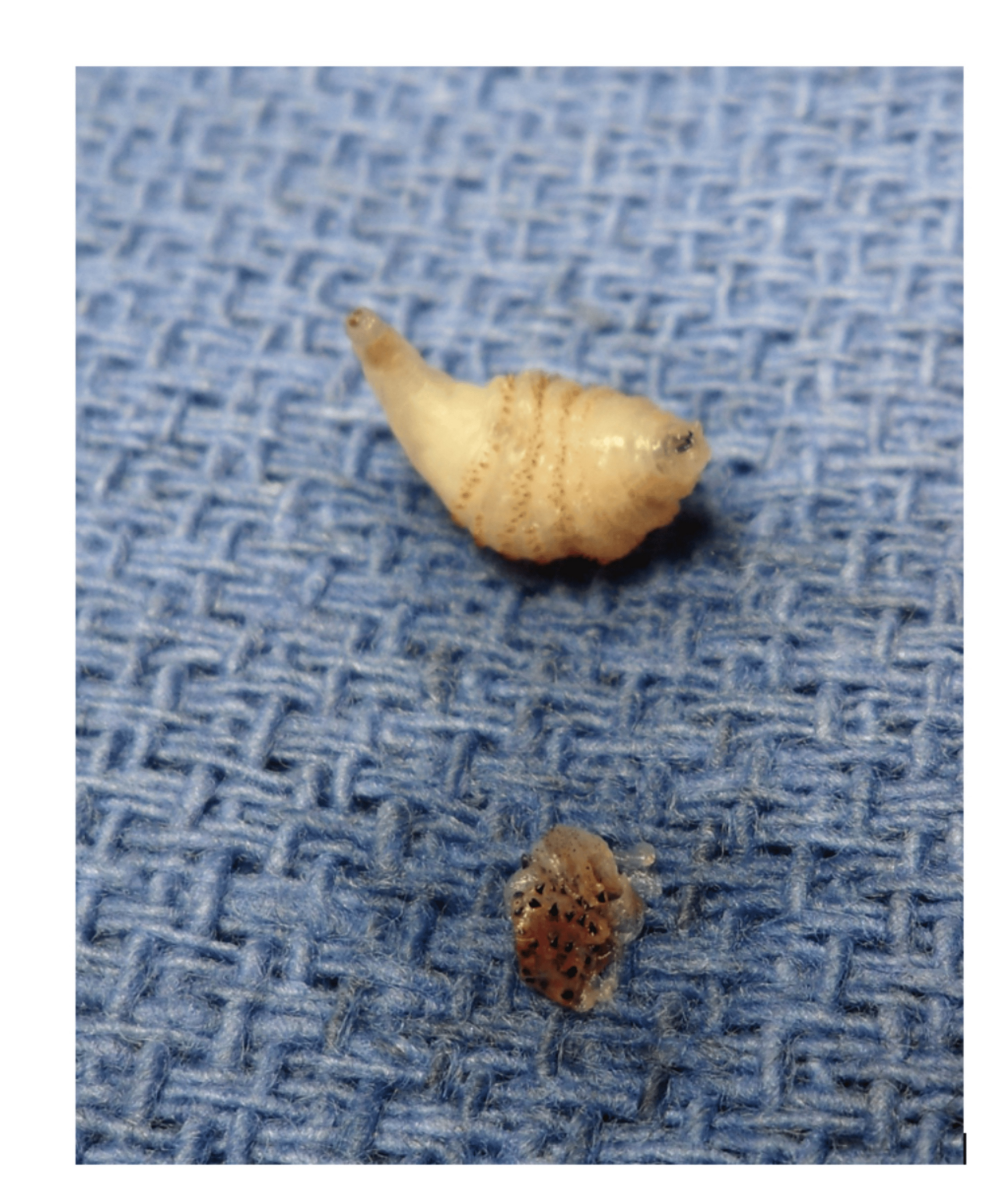
Two intact intraoperative larvae extracted from scalp lesions.

Given the patient’s age and pain, the plastic surgery team took the child to the operating room under general anesthesia for definitive exploration of all lesions. Thick white material was encountered in the subcutaneous cavities, but it was not consistent with frank pus. When the fibrous capsule of one lesion was excised, the material within began to move. This material was identified as a motile larva (Figure 1). All four lesions were surgically incised and explored, and a total of four larvae were extracted. All larvae were removed intact without complication. The wound cavities were irrigated and left to heal by secondary intention, and the patient was discharged. Clindamycin, which the patient had been receiving for two days, was stopped at this time. At a two-week follow-up visit, all lesions had healed completely with minimal scarring and no signs of secondary infection.

## Discussion

The present case illustrates the diagnostic challenge of furuncular myiasis in a pediatric patient outside of endemic regions. The initial clinical presentation, multiple localized swellings on the scalp and arm, was indistinguishable from routine bacterial abscesses. Indeed, the true etiology was only uncovered intraoperatively, illustrating how easily myiasis can be missed if clinicians are unaware of the condition. Travel history was pivotal in retrospect; the patient’s recent trip to the Caribbean provided the key epidemiologic clue. In recent years, frequent international travel has made myiasis an increasingly recognized import in returning travelers, yet misdiagnosis remains common due to limited awareness outside endemic areas [5,6]. A high index of suspicion is required, especially in children, to avoid unnecessary procedures and delays in appropriate treatment.

This case also emphasizes that multiple lesions should not exclude the diagnosis of furuncular myiasis. Although *Dermatobia hominis* classically presents as a solitary lesion, children or heavily exposed individuals may harbor multiple larvae from a single exposure, as demonstrated in prior travel-related reports [1]. In our patient, the presence of four larvae indicates that simultaneous furuncular nodules can occur and should not lead clinicians away from this diagnosis [4].

In travelers, furuncular lesions that do not yield purulent material on attempted drainage should raise suspicion of a parasitic infection. Repeated unsuccessful I&D or "sterile" abscesses are classic red flags for myiasis and warrant careful inspection for a punctum or larval movement, along with a focused travel history. Definitive treatment requires complete larval extraction, and in young children, operative removal under general anesthesia may be necessary to ensure intact removal and minimize inflammatory complications.

Our experience aligns with the literature that emphasizes early recognition of myiasis to prevent mismanagement. In one report, a patient with botfly infestation saw three different physicians and received two courses of antibiotics before the correct diagnosis was made [7]. Similarly, Dermatobia larvae have been mistaken for abscesses or cellulitis and treated as such until surgical exploration revealed the parasite [4]. Increased awareness is needed among pediatric and emergency providers. Had the travel history been obtained earlier in this case, the diagnosis might have been suspected before surgery. Fortunately, once diagnosed, complete removal of the larvae usually results in a cure without the need for prolonged antimicrobials [5].

## Conclusions

Pediatric botfly myiasis can closely mimic common bacterial abscesses, leading to potential misdiagnosis. The present case highlights the importance of maintaining a high index of suspicion for furuncular myiasis in children with non-healing "boils" and a relevant travel history. Early recognition and appropriate intervention (often surgical extraction of the larvae, especially in young patients) are critical to avoid repeated invasive procedures and unnecessary antibiotic exposure. A thorough travel history, combined with awareness of the characteristic clinical signs of *D. hominis* infestation, is key to timely diagnosis and management.
